# Utility of liver and intestinal fatty acid-binding proteins in diagnosing intra-abdominal injury in adult trauma patients: prospective clinical trial

**DOI:** 10.1093/bjs/znac117

**Published:** 2022-05-18

**Authors:** Willem J J de Jong, Mostafa El Moumni, Klaus W Wendt, Maarten W Nijsten, Jan B F Hulscher

**Affiliations:** Department of Surgery, University Medical Centre Groningen, University of Groningen, Groningen, the Netherlands; Department of Surgery, University Medical Centre Groningen, University of Groningen, Groningen, the Netherlands; Department of Surgery, University Medical Centre Groningen, University of Groningen, Groningen, the Netherlands; Department of Critical Care, University Medical Centre Groningen, University of Groningen, Groningen, the Netherlands; Department of Surgery, University Medical Centre Groningen, University of Groningen, Groningen, the Netherlands

## Introduction

Diagnostic evaluation of patients with potential traumatic intra-abdominal injury (IAI) can be time-consuming, expensive^1^, operator-dependent^2^, and potentially harmful owing to radiation exposure or intravenous contrast^3,4^. A specific serum marker for IAI would be a valuable addition to the diagnostic work-up. Fatty acid-binding proteins (FABPs) are small proteins present in various organs, including abdominal organs^5,6^. Liver FABP (L-FABP) is mainly present in the liver and intestinal FABP (I-FABP) mainly in small bowel enterocytes^7^. The measurement of circulating FABPs may be a valuable adjunct to the diagnostic evaluation of abdominal injury^8–14^. This study assessed whether L- and I-FABPs are clinically applicable biomarkers for IAI in a consecutive prospective cohort of patients with polytrauma.

## Methods

### Setting

The University Medical Centre Groningen is a level I trauma centre in the Netherlands. The study was approved by the Medical Ethical Committee of the University Medical Centre Groningen and registered in the Netherlands Trial Register (NTR2211). Informed consent was obtained within 48 h after trauma from either the patient or a patient proxy, using deferred consent^15,16^.

### Patients

All patients aged 18 years or older admitted to the shock room with a suspicion of serious injury were eligible. Patients referred from another hospital were excluded, as were those admitted for longer than 3 h after trauma before inclusion.

All patients were treated according to the Advanced Trauma Life Support guidelines. Ultrasonography of the abdomen was performed in all patients. CT was undertaken as deemed necessary by the attending trauma team. IAI was defined as the presence of injury on CT or by intraoperative findings. IAI was considered as injury to the abdominal gastrointestinal and urogenital tracts, as well as vascular injury; fractures of the pelvis were not. An independent radiologist reviewed all CT images of suspected IAI. The group was subsequently divided into patients with and without IAI.

Blood samples were obtained on arrival in the emergency room, 3 h after injury, and on the first day after trauma. Fifteen healthy volunteers without a medical history served as laboratory controls^17^. Specimens were used for duplicate measurement of L- and I-FABP levels. A commercially available enzyme-linked immunosorbent assay (ELISA) kit (R&D Systems, Abingdon, UK) was used by an analyst blinded to the clinical status.

### Statistical analysis

Owing to lack of data on these markers in injured patients, a sample size was calculated using an effect size of 0.5 and a power of 80 per cent with an α of 0.05. It was calculated that 65 patients would be needed in each group.

Continuous variables were compared using Student’s *t* test or Mann–Whitney *U* test, and categorical data using χ^2^ or Fisher’s exact test. Spearman’s test was used for analysis of non-parametric correlations. *P* < 0.050 was considered statistically significant. Receiver operating characteristic (ROC) curves were generated and the Youden index was used to compute optimal cut-off levels. Multivariable logistic regression analysis was used to assess the independent relationship between I-FABP or L-FABP with IAI. SPSS^®^ version 24 (IBM, Armonk, NY, USA) was used for all analyses.

## Results

### Patient characteristics

Between March 2010 and April 2014, there were 739 eligible patients. A total of 600 patients were included, 123 declined consent, and no blood was withdrawn on admission from 16 patients. Characteristics of the enrolled patients are summarized in *Table 1*. Patient characteristics of those not enrolled did not differ significantly from those of the study group. Twenty-eight IAI patients (44 per cent) had a laparotomy and four (6 per cent) underwent (successful) angioembolization.

**Table 1:** Characteristics of patients with and without abdominal injuries

	Intra-abdominal injury (*n* = 64)	No intra-abdominal injury (*n* = 536)	*P*†
Age (years)*	37 (25–52)	49 (32–65)	<0.001‡
**Men**	47 (73)	405 (76)	0.71
ISS*	29 (16–43)	17 (5–29)	<0.001‡
AIS score*			
Head	1 (0–3)	2 (0–4)	0.54‡
Face	0 (0–1)	0 (0–1)	0.93‡
Thorax	3 (2–4)	0 (0–3)	<0.001‡
Abdomen	3 (2–4)	0 (0–0)	<0.001‡
Extremities	2 (0–3)	0 (0–2)	0.002‡
EMTRAS*	4 (2–5)	3 (2–5)	0.03‡
Mean arterial pressure (mmHg)*	80 (57–97)	93 (83–102)	<0.001‡
Shock index*	0.84 (0.69–1.24)	0.64 (0.52–0.79)	<0.001‡
GCS score*	14 (7–15)	14 (7–15)	0.49‡
**Intubated in ER**	35 (55)	267 (50)	0.46
**Blunt trauma**	59 (92)	511 (95)	0.28
**Trauma mechanism**			0.01
Car	31 (48)	144 (27)	
Fall from height	6 (9)	152 (28)	
Bicycle	7 (11)	65 (12)	
Motorcycle	6 (9)	50 (9)	
Pedestrian	3 (5)	19 (4)	
Violence	4 (6)	21 (4)	
Miscellaneous	7 (12)	85 (16)	
**Intra-abdominal injury specified**			
Liver	26 (41)		
Spleen	21 (33)		
Kidney	19 (30)		
Pancreas	2 (3)		
Gut	15 (23)		
Stomach	2 (3)		
Bladder	4 (6)		
Diaphragm	4 (6)		
Vascular	2 (3)		
Mesocolon	1 (2)		
Duration of hospital stay (days)*	22 (10–32)	8 (2–17)	<0.001‡
Duration of ICU stay (days)*	4 (2–15)	3 (1–9)	0.06‡
**Death in hospital**	14 (22)	103 (19)	0.61

Values in parentheses are percentages unless indicated otherwise; *values are median (i.q.r.). ISS, Injury Severity Score; AIS, Abbreviated Injury Scale; EMTRAS, Emergency Trauma Score; GCS, Glasgow Coma Scale; ER, emergency room. †χ^2^ or Fisher’s exact test, except ‡Mann–Whitney *U* test.

### Relationship between fatty acid-binding proteins and clinical outcome

**Fig. 1:**
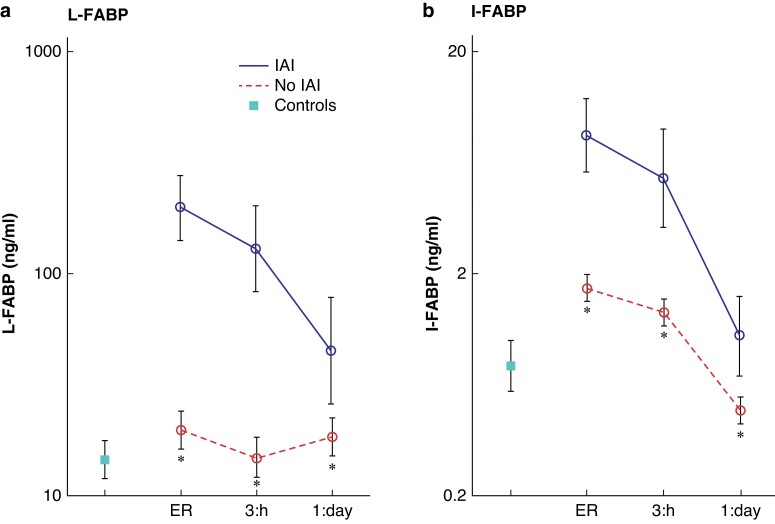
Plasma levels of liver and intestinal fatty acid-binding proteins, in controls and patients with and without abdominal injury, in the emergency room, and 3 h and 1 day after injury **a** Liver fatty acid-binding protein (L-FABP) and **b** intestinal fatty acid-binding protein (I-FABP). Median levels and 95 per cent confidence intervals are shown. IAI, intra-abdominal injury; ER, emergency room. *P < 0.001 *versus* IAI (Mann–Whitney *U* test).

L- and I-FABP levels were significantly higher in the IAI group on admission, and after 3 h and 1 day (*P* < 0.001) (*Fig. 1*). Optimal cut-off points were computed to be 53 ng/ml for L-FABP and 3.5 ng/ml for I-FABP. For L-FABP, the sensitivity was 87 per cent, specificity 62 per cent, and area under the curve 0.82. Positive (LR+) and negative (LR−) likelihood ratios were 2.3 and 0.2 respectively. Negative (NPV) and positive (PPV) predictive values were 97 and 22 per cent respectively. For I-FABP, the sensitivity was 71 per cent, specificity 74 per cent, and the area under the curve 0.77. LR+ was 2.7 and LR− was 0.4. NPV was 95 per cent and PPV was 25 per cent. *Table 2* shows correlations between L-FABP or I-FABP and clinical outcome parameters.

**Table 2:** Correlation between liver and intestinal fatty acid-binding protein levels and clinical parameters on admission to the emergency department

	L-FABP		I-FABP	
	Correlation coefficient	*P*	Correlation coefficient	*P*
**Age**	0.01	0.84	0.05	0.23
**ISS**	0.28	<0.001	0.37	<0.001
**AIS—abdomen**	0.26	<0.001	0.30	<0.001
**EMTRAS**	0.23	<0.001	0.37	<0.001
**GCS score**	–0.12	0.01	−0.24	<0.001
**Shock index**	0.26	<0.001	0.36	<0.001
**Duration of hospital stay**	0.13	<0.001	0.20	<0.001
**Duration of ICU stay**	0.12	0.03	0.14	0.01

L-FABP, liver fatty acid-binding protein; I-FABP, intestinal fatty acid-binding protein; ISS, Injury Severity Score; AIS, Abbreviated Injury Scale; EMTRAS, Emergency Trauma Score; GCS, Glasgow Coma Scale.

Median L- and I-FABP levels on admission in patients with abdominal injuries requiring intervention and those treated conservatively were 81.0 *versus* 263.4 ng/ml (*P* = 0.103) and 8.9 *versus* 7.3 ng/ml (*P* = 0.275) respectively.

The 10 patients with the highest I- and L-FABP levels without IAI all sustained severe traumatic brain injury (Abbreviated Injury Scale score over 5).

Logistic regression analysis showed that L- and I-FABPs were independently prognostic for the presence of IAI when overall injury severity (Injury Severity Score) was taken into account (*Table S1*).

### Missed injury

In one patient, transection of the small bowel was missed during the initial evaluation, which included abdominal CT. The patient underwent laparotomy 36 h after trauma. L- and I-FABP levels on admission were 794 and 61 ng/ml respectively.

## Discussion

These results suggest that L- and I-FABPs are clinically useful biomarkers for IAI in injured adult patients, and that L-FABP is slightly superior to I-FABP in ruling out IAI. Only injuries detected by CT or during laparotomy were considered IAI, this may have caused a number of false-negative results and thus lowered the measured predictive power of the biomarkers. A remarkable finding on assessment of patients with increased L- and I-FABP levels, but without IAI, was that the majority had severe traumatic brain injury. L- and I-FABPs are organ-specific, and are not present in cells of the brain. However, patients with severe traumatic brain injury, especially those who die as a result of these injuries, undergo a cytokine storm^18^. The increase in L- and I-FABP levels in these patients may therefore be the result of this overwhelming inflammatory response causing damage to cells throughout the body.

Median L-FABP levels in patients with IAI who were treated conservatively were three times higher than levels among than those requiring intervention. Although not statistically significant, this is remarkable and probably due to the release of L-FABP from liver injuries not requiring intervention, whereas patients requiring intervention more often sustained small bowel/colon injuries.

A limitation is the time needed to perform the ELISA to obtain the plasma concentrations. Point-of-care tests are, however, available for other isoforms (Heart-FABP), but not yet for L- and I-FABPs. On the other hand, CT is time-consuming and exposes the patient to radiation. L-FABP may aid in decision-making to rationalize use of CT and thereby save time, resources, and radiation exposure. High levels of L- and I-FABPs in patients without abnormal CT findings may warrant closer follow-up as IAI may be present.

## Supplementary Material

znac117_Supplementary_Data
