# Supplementary material for: Utility of liver and intestinal fatty acid-binding proteins in diagnosing intra-abdominal injury in adult trauma patients: prospective clinical trial
Source: Br J Surg. 2022 May 18;109(9):796–9. doi: 10.1093/bjs/znac117 (PMC10364720; doi:10.1093/bjs/znac117)
Supplement: znac117_Supplementary_Data [file znac117_supplementary_data.zip › Supplementary_Table_1.docx]

|  | Intra-abdominal injury | No intra-abdominal injury | p-value |
| --- | --- | --- | --- |
| N | 64 | 536 |  |
| Age (years) | 37 (25-52) | 49 (32-65) | <0.001 |
| Sex (male) | 47 (73%) | 405 (76%) | 0.71 |
| ISS  AIS  Head  Face  Thorax | 29 (16-43)  1 (0-3)  0 (0-1)  3 (2-4) | 17 (5-29)  2 (0-4)  0 (0-1)  0 (0-3) | <0.001  0.54  0.93  <0.001 |
| Abdomen  Extremities | 3 (2-4)  2 (0-3) | 0 (0-0)  0 (0-2) | <0.001  0.002 |
| EMTRAS | 4 (2-5) | 3 (2-5) | 0.03 |
| MAP  Shock index  GCS  Intubated ER | 80 (57-97)  0.84 (0.69-1.24)  14 (7-15)  35 (55%) | 93 (83-102)  0.64 (0.52-0.79)  14 (7-15)  267 (50%) | <0.001  <0.001  0.49  0.46 |
| Blunt trauma  Trauma mechanism  Car  Fall from height  Bike  Motorcycle  Pedestrian  Violence  Miscellaneous | 59 (92%)  31 (48%)  6 (9%)  7 (11%)  6 (9%)  3 (5%)  4 (6%)  7 (12%) | 511 (95%)  144 (27%)  152 (28%)  65 (12%)  50 (9%)  19 (4%)  21 (4%)  85 (16%) | 0.28  0.01 |
| IAI specified:  Liver  Spleen  Kidney  Pancreas  Gut  Gastric  Bladder  Diaphragm  Vascular  Mesocolon  Hospital LOS | 26 (41%)  21 (33%)  19 (30%)  2 (3%)  15 (23%)  2 (3%)  4 (6%)  4 (6%)  2 (3%)  1 (2%)  22 (10-32) | 8 (2-17) | <0.001 |
| ICU LOS | 4 (2-15) | 3 (1-9) | 0.06 |
| Hospital-Mortality | 14 (22%) | 103 (19%) | 0.61 |
