# Supplementary material for: Utility of liver and intestinal fatty acid-binding proteins in diagnosing intra-abdominal injury in adult trauma patients: prospective clinical trial
Source: Br J Surg. 2022 May 18;109(9):796–9. doi: 10.1093/bjs/znac117 (PMC10364720; doi:10.1093/bjs/znac117)
Supplement: znac117_Supplementary_Data [file znac117_supplementary_data.zip › Supplementary_Table_2.docx]

| T_A_ | L-FABP | | I-FABP | |
| --- | --- | --- | --- | --- |
|  | Correlation coefficient | p-value | Correlation coefficient | p-value |
| Age | 0.01 | 0.84 | 0.05 | 0.23 |
| ISS | 0.28 | <0.001 | 0.37 | <0.001 |
| AIS-abdomen  EMTRAS | 0.26  0.23 | <0.001  <0.001 | 0.30  0.37 | <0.001  <0.001 |
| GCS | -0.12 | 0.01 | -0.24 | <0.001 |
| Shock index | 0.26 | <0.001 | 0.36 | <0.001 |
| Hospital-LOS | 0.13 | <0.001 | 0.20 | <0.001 |
| ICU-LOS | 0.12 | 0.03 | 0.14 | 0.01 |
