# Supplementary material for: Utility of liver and intestinal fatty acid-binding proteins in diagnosing intra-abdominal injury in adult trauma patients: prospective clinical trial
Source: Br J Surg. 2022 May 18;109(9):796–9. doi: 10.1093/bjs/znac117 (PMC10364720; doi:10.1093/bjs/znac117)
Supplement: znac117_Supplementary_Data [file znac117_supplementary_data.zip › Supplementary_Table_3.docx]

| **Variable** | **OR (95% confidence interval)** | **p-value** |
| --- | --- | --- |
| L-FABP at T_A_ | 1.002 (1.001 – 1.003) | <0.001 |
| ISS | 1.036 (1.018 – 1.055) | <0.001 |
|  |  |  |
| I-FABP at T_A_ | 1.020 (1.007 – 1.033) | 0.002 |
| ISS | 1.040 (1.022 – 1.058) | <0.001 |
